# Integrating livestock and cropping systems: Interseeding cereal rye into corn for grazing

**DOI:** 10.1002/jeq2.70026

**Published:** 2025-04-25

**Authors:** Kathy J. Soder, Paul R. Adler, Curtis J. Dell, Benjamin C. Williamson

**Affiliations:** ^1^ USDA‐ARS Pasture Systems and Watershed Management Research Unit University Park Pennsylvania USA; ^2^ Department of Animal Science The Pennsylvania State University University Park Pennsylvania USA

## Abstract

Interseeding annual forages into growing corn may be an alternative for both cover and grazing in temperate regions of the United States. A 4‐year experiment evaluated the effect of interseeding cereal rye (*Secale cereale*) into corn for grazing after harvest on corn grain, forage biomass yield and quality, soil health, and estimated spatial biomass yields using vegetation indices (VIs) from multispectral imagery collected from an unmanned aircraft system platform. Corn was planted (79,074 plants ha^−1^) each spring on two 4.8‐ha sites in central Pennsylvania. Cereal rye was interseeded (135 kg ha^−1^) into corn at the V4–V6 stage. Corn was harvested as grain in November, and each site was subdivided and randomly assigned to grazed or non‐grazed treatments. Biomass yield and quality, soil samples, and estimates of biomass yield using VIs from multispectral imagery monitoring occurred in fall and spring. Results indicated that cereal rye plus corn stover provided enough forage for an additional 105–130 animal unit days ha^−1^ with minimal impact on soil health indicators. Vegetative indices varied in the ability to predict biomass yield; all VIs except normalized difference red edge saturated at ∼2 Mg ha^−1^. Spring growth of cereal rye was much less dependable than fall. Corn grain yields did not decrease (averaging 9.9 tonnes of dry matter ha^−1^) as a result of grazing or due to continuous corn planting except in 2019 (dry year) when corn grain yields were reduced by 35%–40%. Interseeding cereal rye into corn that is harvested as grain can be a viable method to establish a cover crop to extend the grazing season without impairing cash crop yield.

## Core Ideas

- Interseeding cereal rye into growing corn can provide cover crop and grazing opportunities.
- Grazing cereal rye provided 105–130 animal unit days ha^−1^ in late fall without impairing corn grain yield.
- Soil health indicators were only minimally impacted by grazing.
- Using an unmanned aircraft system to characterize spatial forage biomass yields showed promise for pasture management.
- Grazing cover crops can extend the grazing season without impairing cash crop yields.

**Abbreviations**
B: blue
CP: crude protein
DM: dry matter
EVI: enhanced vegetation index
FA: fatty acids
GNDVI: green normalized difference vegetation index
GPS: global positioning system
GRAZ: grazed
NDF: neutral detergent fiber
NDFD: NDF digestibility
NDFDp: predicted NDFD
NDFn: nitrogen‐free NDF
NDRE: normalized difference red edge
NDVI: normalized difference vegetation index
NFC: nonfibrous carbohydrates
NG
: non‐grazed
NIRS: near‐infrared spectroscopy
OSAVI: optimized soil‐adjusted vegetation index
R: red
RE: red‐edge
TDN: total digestible nutrients
UAN: urea ammonium nitrate
UAS: unmanned aircraft system
VI: vegetation index

## 1 INTRODUCTION

Harvesting corn (*Zea mays*) as grain in late fall typically does not provide opportunity for a cover crop to be established in many temperate regions, resulting in increased potential for nutrient leaching and soil erosion (Nelson, 2002). Cool‐season annual forages interseeded into growing corn can provide an established cover crop after corn harvest to improve soil retention over the winter (Antosh et al., 2022; Curran et al., 2018; Liebig et al., 2015). Cover crops were initially planted for conservation benefits (Adetunji et al., 2020). However, with feed costs comprising >50% of operational costs on livestock farms (USDA‐ERS, 2025), grazing these cover crops may provide opportunity to utilize this additional forage and reduce stored feed costs (Tobin et al., 2020). Finding strategies to extend the grazing season beyond the traditional growing season could substantially decrease feed costs, thereby improving farm profitability (Poore et al., 2000). Many farmers are reluctant to plant or grazed (GRAZ) cover crops due to perceived concerns about significant cost with no immediate economic benefit, soil damage from animal hooves, and reduced ground cover after grazing (Hayden et al., 2018; Roesch‐McNally et al., 2017). There is great opportunity to combine the need for low‐cost feed along with the conservation benefits of cover crops into a novel approach to increase the overall economic and environmental sustainability of small farms.

Winter cover crops have generally been shown to increase soil organic carbon in corn production systems, although the amount of organic matter added has varied depending on several factors such as soil properties, cover crop species, tillage, and weather variability (Joshi et al., 2023; McClelland et al., 2020). The accumulation of soil organic matter with cover crop production has often been associated with improvement in soil biological, physical, and chemical properties, enhancing nutrient and water retention and improving soil structure (Hao et al., 2023). However, grazing could potentially offset some of the benefits of the cover crops. A review by Faust et al. (2017) points out that hoof traffic during grazing increases the potential for soil compaction and losses of sediments and nutrients in runoff water but the severity of the impact is greatly influenced by stocking rate and the frequency and timing of grazing. Grazing can also modify the distribution of C and nutrient inputs within a field, as the majority of the C and nutrients in forage consumed during grazing are concentrated and unevenly distributed with dung and urine deposition (Faust et al., 2017). This uneven distribution of nutrients then potentially alters nutrient availability to subsequent crops and can increase potential for N leaching from urine patches. Studies with grain production systems on the US Great Plains and southern Piedmont suggest soil health and water quality benefits of cover crops can largely be retained with well‐managed grazing (Faust et al., 2017; Franzluebbers & Stuedemann, 2008; Kelly et al., 2021; Simon et al., 2024; Singh et al., 2022). However, impacts on winter cover crops have not been reported for the northeastern United States.

Remote sensing has potential to characterize the spatial distribution of forage biomass and quality and, therefore, may be used as a tool to aid in directing precision grazing strategies (Bretas et al., 2023). Unmanned aircraft systems (UASs) have been used to characterize biomass yield of crops including grasslands (Bazzo et al., 2023; Chen et al., 2021; Théau et al., 2021) and cover crops (J. Miller et al., 2024; Prabhakara et al., 2015; Yuan et al., 2019). Common strategies of biomass yield estimation include volume measurement using structure‐from‐motion photogrammetry (Cunliffe et al., 2016; Théau et al., 2021) and digital surface models (J. Miller et al., 2024), and vegetation indices (VIs) such as normalized difference vegetation index (NDVI) and normalized difference red edge (NDRE), green normalized difference vegetation index (GNDVI), enhanced vegetation index (EVI), and optimized soil‐adjusted vegetation index (OSAVI; J. Miller et al., 2024; Prabhakara et al., 2015; Yeom et al., 2019).

Grazing cover crops interseeded into cash crops could provide added revenues and increase nutrient cycling in the system while also providing ecosystem services that can affect long‐term productivity and economics. However, the trade‐offs between cash crop productivity and environmental effects are not fully understood. Therefore, the objective of this 4‐year study was to evaluate the effects of interseeding cereal rye (*Secale cereale*) into corn for grazing after corn grain harvest on corn grain and forage biomass yield and quality, soil health, and estimated spatial biomass yields using VIs from multispectral imagery collected from a UAS. We hypothesized that grazing the cover crop would have no negative impact on corn grain yields compared to not grazing the cover crop but would extend the grazing season to decrease stored forage needs.

## 2 MATERIALS AND METHODS

### 2.1 Site description

The study was conducted on two 4.8‐ha sites located approximately 0.5 km apart at the Russell E. Larson Agricultural Center, Rock Springs, PA (40°43′00″ N, 77°56′24″ W) from 2018 through 2021. Soil at one site is primarily Hagerstown silt loam with slopes of 3%–8% (fine, mixed, semiactive, mesic Typic Hapludalfs), and soil at the second site is primarily Murrill channery silt loam (fine‐loamy, mixed, semiactive, mesic Typic Hapludalfs) with slopes of 3%–8%. A detailed description of topography and climate can be found in Soder et al. (2024). The study was initiated in 2016. In 2016 and 2017, annual ryegrass (*Lolium multiflorum*) was used as the cover crop. Corn planting and harvest, and grazing management were identical in 2016–2017 to what is described in the current study. Due to poor establishment and late spring emergence of the annual ryegrass used (non‐determinant variety), it was decided to use cereal rye instead starting in 2018. Only data from 2018 to 2021 are reported in this paper. Two weeks prior to corn planting in 2018 and thereafter, both sites were sprayed with 2.34 L ha^−1^ glyphosate (N‐(phosphonomethyl)glycine) and 74 mL ha^−1^ saflufenacil (30%) with lambda‐cyhalothrin (12%; 1 L 100 L^−1^) added as an adjuvant to control existing vegetation. Both sites were no‐till planted with 98‐day relative maturity corn (79,074 plants ac^−1^) in May each year. At corn planting, 5.2 kg N ha^−1^ and 7.7 kg P ha^−1^ as ammonium phosphate (10‐34‐0) was applied with seed, and 37 kg N ha^−1^ was applied 5 cm to the side and 5 cm below the seed as urea ammonium nitrate (UAN, 30%). Two weeks prior to interseeding cereal rye, both sites were sprayed with 2.34 L ha^−1^ glyphosate (N‐(phosphonomethyl)glycine) and 2.34 L ha^−1^ glufosinate‐ammonium to suppress weeds. Cereal rye was interseeded (135 kg ha^−1^) with an Interseeder planter (Interseeder Technologies, LLC) when corn reached the V4–V6 stage. A dribble band application of 153 kg N ha^−1^ as UAN occurred at the time of interseeding.

### 2.2 Grazing and forage management

**FIGURE 1:**
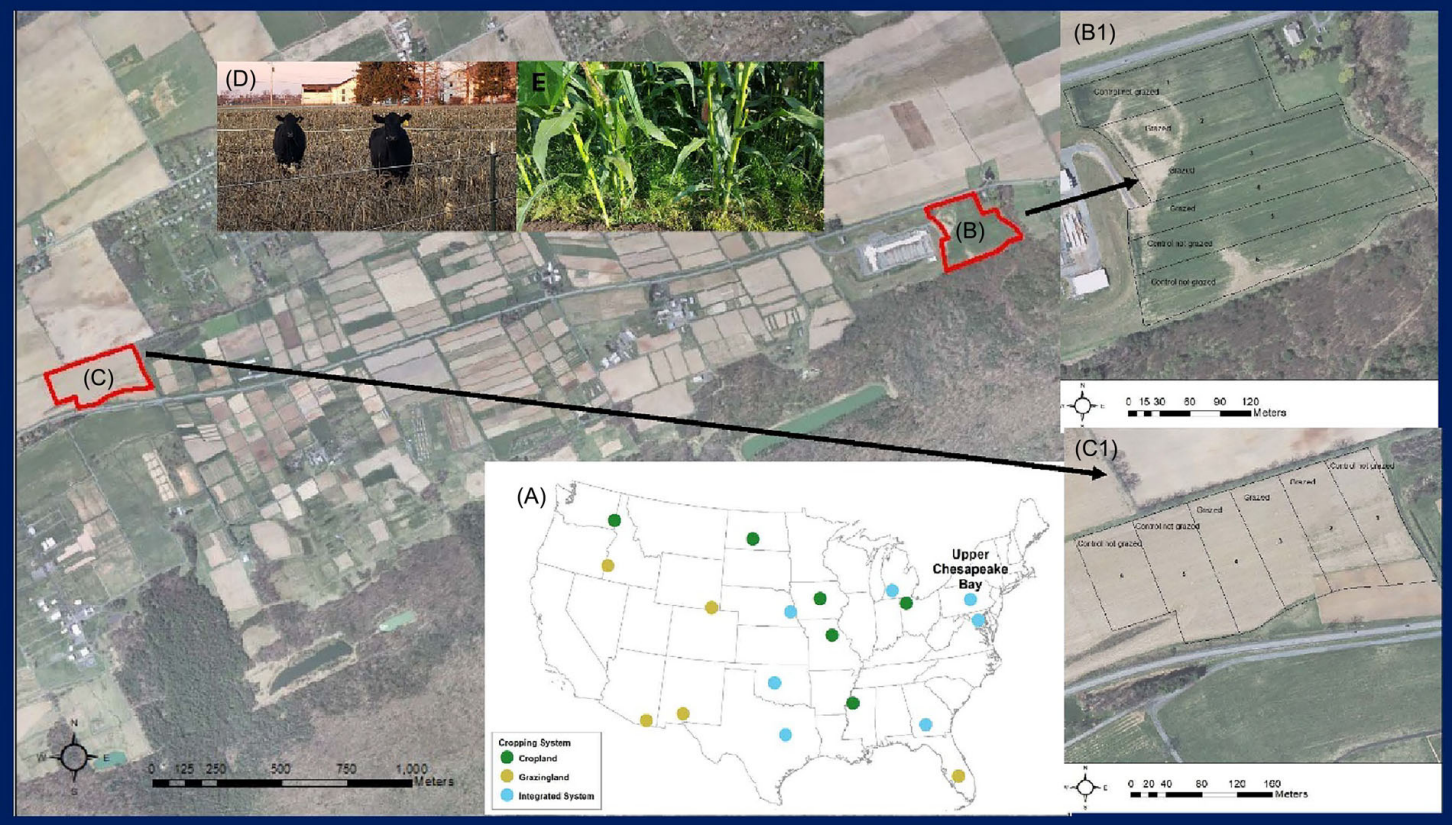
Map of Long‐Term Agroecosystem Research (LTAR) network showing the location of the Upper Chesapeake Bay (UCB) site (A). Location of replicated sites (B and C) for prevailing (non‐grazed) and alternative (grazed) treatments, with expanded view of non‐grazed and grazed paddocks (B1 and C1) in the UCB LTAR Integrated Common Experiment. Pictures of beef cows grazing corn stubble and cereal rye in autumn (D) and cereal rye interseeded into corn (E). *Source*: Photo credits (D and E): Kathy Soder, USDA‐ARS.

Each site was divided into six 0.8‐ha paddocks at the beginning of the study and randomly assigned one of two treatments (GRAZ or non‐grazed [NG]). Paddock layout can be seen in Figure 1. Within each GRAZ paddock, temporary electric fence was used to contain animals. Sub‐paddocks were created with temporary electric fence to provide 2–3 days of available forage (0.25 ha sub‐paddock^−1^). Twenty‐four beef cows (450 kg body weight) were stocked at four cows paddock^−1^ and rotationally grazed on corn stover/cereal rye in each of the six GRAZ paddocks (three of the six paddocks at each site) starting in mid‐ to late‐November. This resulted in a stocking density of 16 animal units ha^−1^. Each sub‐paddock was grazed for 2–3 days until corn stubble and cereal rye were consumed or trampled. No back fencing was used due to lack of additional forage biomass growth and the need to return to a centralized water source. Cows were checked at least twice daily, had unlimited access to fresh water, and were managed under a Penn State University Institutional Animal Care and Use Committee protocol (#PROTO202200411).

**Core Ideas**

- Interseeding cereal rye into growing corn can provide cover crop and grazing opportunities.
- Grazing cereal rye provided 105–130 animal unit days ha^−1^ in late fall without impairing corn grain yield.
- Soil health indicators were only minimally impacted by grazing.
- Using an unmanned aircraft system to characterize spatial forage biomass yields showed promise for pasture management.
- Grazing cover crops can extend the grazing season without impairing cash crop yields.

If spring green‐up was early enough the following year, 24 beef cows from the same herd grazed the cereal rye in the same GRAZ paddocks at the same stocking density (four cows paddock^−1^) as in the fall, starting in early‐ to mid‐April until cereal rye was consumed or trampled, or until the cows had to be removed for preparation for corn planting, whichever came first.

### 2.3 Measurements

Daily air temperature and precipitation data were monitored by a weather station located within 2 km of each site. Summarized monthly weather data can be found in Soder et al. (2024). Precipitation and soil moisture data for the periods when the cattle grazed can be found in Figures S1 and S2.

Immediately before corn grain harvest in late October/early November, whole plant biomass yield was evaluated by cutting two 2.25‐m long rows of corn stalks paddock^−1^ (GRAZ and NG; approximately 60 stalks row^−1^) at ground level and weighed. Corn ears were then removed for hand shelling. Six corn stalks (with ears removed) from each paddock were randomly selected, six shelled corn cobs were added to the stalks, and stalks (with cobs) were then weighed and dried at 60°C for 3 days for dry matter (DM) determination. A 0.75‐L subsample of grain from each paddock was moisture tested with a hand‐held meter, then dried at 60°C for 48 h for DM determination. Corn grain was mechanically harvested with a commercial corn harvester. Corn grain weights were recorded for each paddock.

One day prior to initiation of grazing in fall (approximately 3–4 weeks after corn harvest) and spring (approximately 2–3 weeks after spring green‐up), 20 pre‐graze forage heights were collected from random locations in the first sub‐paddock to be grazed (or not grazed) from all paddocks (GRAZ and NG, both sites) using a meter stick. Four 1‐m^2^ quadrats in each of the six paddocks (GRAZ and NG) site^−1^ were positioned to include three rows of corn and marked via global positioning system (GPS) for UAS imagery collection. Quadrats were clipped to ground level for pre‐grazing forage biomass availability of corn residue and cereal rye. Clipped biomass was separated by species, weighed, oven‐dried at 60°C for 5 days, and weighed for DM yield. Quadrats within each paddock (GRAZ and NG) within site were composited for nutritive analysis. Pre‐graze quadrat sampling was repeated on the last sub‐paddock (but not at every sub‐paddock shift) as described above.

After cattle were removed in fall and spring, four 1‐m^2^ quadrats paddock^−1^ (GRAZ and NG) near the GPS points were collected near the pre‐graze quadrat sites and processed as described above for post‐grazing forage biomass availability (corn residue and cereal rye separately) and forage quality (composited sample). If grazing did not occur in the spring (2019 and 2020), post‐grazing samples were not collected. Corn residue was not collected during any spring samplings (if present) as it would be degraded and not consumed by cows. In Spring 2021, there was no post‐grazing residue; therefore, quadrats were not collected. After spring grazing (2021 only) or when corn planting needed to occur when spring grazing did not occur due to lack of sufficient forage biomass (2019 and 2020), all paddocks on both sites were mowed (with no additional removal of forage) and sprayed as stated above to kill residual forage in preparation for corn planting.

All dried samples were first ground using a Retsch SM 400XL floor grinder (Retsch) with no sieve and then reground with a 20‐mm sieve. The samples were then ground to 1 mm using a Thomas Model 4 Wiley mill (Thomas Scientific). Each sample was thoroughly mixed and subsampled by hand, then ground to 1 mm using a Retsch Twister cyclone mill (Retsch) in preparation for nutritive analysis via near‐infrared spectroscopy (NIRS; McIntosh et al., 2022). Samples were analyzed for concentrations of DM, crude protein (CP), acid detergent fiber, neutral detergent fiber (NDF), and 48‐h in vitro DM digestibility using the 2022 Grass Hay calibration equations and the 2022 Mixed Hay calibration equations, as provided by the NIRS Forage and Feed Testing Consortium (NIRSC, 2020). Samples were analyzed using a Foss DS3F NIRS (Foss Analytical) that was standardized to the NIRS Consortium master instrument to ensure prediction accuracy. ISIscan Nova software was used for NIRS analysis. Nutritive value data are reported with predictions fitting the allowable *H* < 3.0 (Murray & Cowe, 2004). Total digestible nutrients (TDN) were calculated using the NIRS output from using the following equation (NRC, 2001):

$$\begin{array}{ccc} \mathrm{TDN} & = & \left(\mathrm{NFC}\times 0.98)+(\mathrm{CP}\times 0.87)+(\mathrm{FA}\times 0.97\times 2.25\right)\\ & & +\;(\mathrm{NDFn}\times \left[\mathrm{NDFDp}/100\right])-10 \end{array}$$

All values used in the calculation of TDN come from the NIRS analysis, where NDFD is 48‐h in vitro digestibility (% of NDF); FA is fatty acids, FA = ether extract − 1 (% of DM); NFC is nonfibrous carbohydrates, NFC = 1 − (NDFn + CP + Fat + ash); NDFn is nitrogen‐free NDF, NDFn = NDF × 0.93; and NDFDp is predicted NDFD, NDFDp = 22.7 + 0.664 × NDFD (Undersander et al., 2010). A subset (15%) of samples were randomly selected from each paddock and harvested for validation of nutritive value parameters. Samples were analyzed for CP (AOAC, 2000) and NDF (Van Soest et al., 1991) at Cumberland Valley Analytical Services. The standard error of prediction for CP was 1.14 and for ADF was 3.63, while the coefficient of determination (*R* ^2^) for cross validation was 0.96 and 0.95, respectively.

While soil samples were not obtained at the beginning of the study, soil samples were obtained prior to corn planting in the spring of 2021 for evaluation of soil health indicators. Sixteen 5‐cm diameter by 15‐cm deep cores were obtained manually with a drop‐hammer corer from each paddock. Samples were evenly spaced across the paddock and taken between cover crop rows. Dung piles, hoof marks, and other evidence of grazing were not readily detectable at the time of sampling and not accounted for in sampling pattern. Field‐moist samples were passed through an 8‐mm sieve, soil from all cores from a paddock combined, soil thoroughly mixed, and a subsample (approximately 1 L) from each paddock was shipped to the Cornell Soil Health Laboratory for analysis. Available water content, aggregate stability, organic matter, organic C, total C, total N, extractable protein index, respiration rate, active C, pH, and Mehlich‐3 extractable nutrients (P, K, Ca, Mg, S, Al, B, Cu, Fe, Mn, and Zn) were determined. Analytical methods used can be found in Moebius‐Clune et al. (2017). Because there was no detectable inorganic C in the soils at the sites, total soil C pool was assumed to be all organic C, and values for total soil C were not reported.

Multispectral imagery was collected before grazing in the fall (November 2020) and spring (April 2021) using a DJI Matrice 210 RTK V2 equipped with a Micasense RedEdge‐M multispectral sensor. A 75% side and 85% front overlap was used for flights 65 m above ground level, providing a 4.5‐cm pixel resolution. Following each flight, post‐processing of the data included radiometric and geometric corrections using photogrammetry software Pix4Dmapper. To apply radiometric corrections, a calibrated Micasense reflectance panel was photographed prior to each flight. Geo‐referenced ground control points were used to correct for spatial location. The following VIs were evaluated to characterize biomass yield and calculated as follows: red (R), blue (B), red‐edge (RE), NIRS: NDVI (NIRS − R)/(NIRS + R), NDRE (RE − R)/(RE + R), EVI 2.5(NIRS − R)/(NIRS + 6 × R − 7.5 × B +1), and OSAVI (NIRS − R)/(NIRS + R+ 0.16).

### 2.4 Statistical analysis

A randomized complete block experimental design with within‐site replication (*n* = 3) was used in this study. Forage biomass, and corn grain yield and quality data were analyzed with a mixed model analysis of variance using PROC MIXED (SAS Institute). Site, grazing treatment, and the interaction of site and grazing treatment were considered fixed effects. Year was considered a random effect. The normality of the data was tested before running any statistical analysis using PROC UNIVARIATE in SAS software, and the Shapiro–Wilk *W* test for normality was used (Shapiro & Wilk, 1965). Data were log or square‐root transformed to achieve normality and variance homogeneity if required. Variation of means was documented using standard error. There were no site main effects or site‐by‐treatment interactions; therefore, means are presented as an average across both sites (Table 1).

**TABLE 1:** Fall pre‐ and post‐grazing corn stover and interseeded cereal rye aboveground dry matter (DM) forage biomass (±standard error) for grazed (GRAZ) and non‐grazed (NG) paddocks (averaged across both sites).

	Year			
Item	Fall 2018	Fall 2019	Fall 2020	Fall 2021
Pre‐graze biomass, tonnes of DM ha^−1^				
GRAZ paddocks				
Corn stover	5.93 ± 0.49	4.92 ± 0.44	5.14 ± 0.47	5.14 ± 0.49
Cereal rye	0.51 ± 0.03c	0.42 ± 0.02a	0.72 ± 0.05b	0.77 ± 0.05b
Total biomass	6.44 ± 0.42	5.34 ± 0.35	5.86 ± 0.17	5.91 ± 0.40
NG paddocks				
Corn stover	5.38 ± 0.37	4.92 ± 0.49	5.71 ± 0.49	5.06 ± 0.57
Cereal rye	0.53 ± 0.02a	0.42 ± 0.02a	0.82 ± 0.07b	0.84 ± 0.10b
Total biomass	5.91 ± 0.35ab	5.34 ± 0.47a	6.53 ± 0.43b	5.90 ± 0.38ab
Grazed	Yes	Yes	Yes	No
Date cows started grazing	November 17, 2018	November 10, 2019	November 16, 2020	–
No. days grazed	26	24	23	–
Post‐graze biomass, tonnes of DM ha^−1^				
GRAZ paddocks				
Corn stover	2.37 ± 0.32A	2.10 ± 0.25A	2.49 ± 0.20A	–
Cereal rye	0.29 ± 0.03cA	0.20 ± 0.02aA	0.37 ± 0.02bA	–
Total biomass	2.56 ± 0.27A	2.30 ± 0.23A	2.86 ± 0.25A	–
NG paddocks				
Corn stover	5.51 ± 0.54B	4.99 ± 0.62B	5.85 ± 0.67B	–
Cereal rye	0.96 ± 0.07B	0.84 ± 0.05B	1.01 ± 0.12B	–
Total biomass	6.47 ± 0.42B	5.83 ± 0.52B	6.86 ± 0.62B	–

*Note*: Lowercase letters within each row with the same letter are not significantly different (*p* < 0.05). Uppercase letters in columns between GRAZ and NG treatments within year and sampling time (pre‐ or post‐graze) with the same letter are not significantly different (*p* < 0.05).

## 3 RESULTS AND DISCUSSION

### 3.1 Biomass yield

Fall pre‐grazing corn stover biomass yields were similar across both treatments (GRAZ and NG) and all years (Table 1). This is not surprising given that all paddocks were planted at the same density of corn and harvested to the same residual height. Fall pre‐grazing cereal rye biomass yields were greatest (*p *< 0.05) in Fall 2018 and least in Fall 2019. Pre‐grazing cereal rye biomass yields were similar between GRAZ and NG paddocks with the exception of Fall 2018 when pre‐grazing cereal rye biomass was greater (*p* < 0.05) for the GRAZ treatment. This also resulted in total biomass being greater in the GRAZ treatment in 2018.

Paddocks were grazed each fall of the study with the exception of 2021, as corn was not harvested until January 2022 due to wet weather (see Soder et al., 2024). For the years when grazing did occur (Fall 2018–2020) in the GRAZ paddocks, corn stover biomass was reduced (*p* < 0.05) by an average of 57% (range of 52%–60%) between pre‐ and post‐grazing samples, with no change in the NG paddocks (Table 1). In the GRAZ paddocks, post‐grazing cereal rye biomass and total biomass yields decreased (*p* < 0.05) by an average of 63% and 57% after grazing, respectively, across years (ranges of 55%–72% and 52%–61%, respectively). The only change in pre‐ to post‐grazing samplings in the NG paddocks was in Fall 2018, where cereal rye and total biomass yields increased by 191% and 13%, respectively, due to the uncharacteristically warmer temperatures (see Soder et al., 2024).

The only year that spring grazing occurred was in 2021 due to limited cereal rye biomass in Spring 2019 and 2020 (Table 2). There was no measurable corn stover residue in spring of any year as it had either been consumed, trampled by cattle, degraded, or crushed by snow/rain over the winter. Cereal rye and total biomass yield was greatest (*p* < 0.05) in Spring 2021 and least in Spring 2020. In Spring 2021, cereal rye biomass decreased by 73% between pre‐ and post‐grazing in the GRAZ paddocks. In the NG paddocks, Spring 2021 cereal rye biomass increased by 28% during the period when cows grazed the GRAZ paddocks.

**TABLE 2:** Spring pre‐ and post‐grazing corn stover and interseeded cereal rye aboveground dry matter (DM) forage biomass (±standard error) for grazed (GRAZ) and non‐grazed (NG) paddocks (averaged across both sites).

	Year		
Item	Spring 2019	Spring 2020	Spring 2021
Pre‐graze biomass, tonnes of DM ha^−1^			
GRAZ paddocks			
Corn stover	0	0	0
Cereal rye	0.44 ± 0.02b	0.07 ± 0.02a	1.01 ± 0.07c
Total biomass	0.44 ± 0.02b	0.07 ± 0.02a	1.01 ± 0.07c
NG paddocks			
Corn stover	0	0	0
Cereal rye	0.42 ± 0.02b	0.10 ± 0.02a	0.87 ± 0.07c
Total biomass	0.42 ± 0.02b	0.10 ± 0.02a	0.87 ± 0.07c
Grazed	No	No	Yes
Date cows started grazing	–	–	April 29, 2021
No. of days grazed	–	–	21
Post‐graze biomass, tonnes of DM ha^−1^			
GRAZ paddocks			
Corn stover	0	0	0
Cereal rye	–	–	0^a^
Total biomass	–	–	0^a^
NG paddocks	–	–	
Corn stover	0	0	0
Cereal rye	–	–	1.11 ± 0.12
Total biomass	–	–	1.11 ± 0.12

*Note*: Lowercase letters within each row with the same letter are not significantly different (*p* < 0.05).

^a^ No material available to harvest after cows grazed.

Paddocks were not grazed in Spring 2019 or 2020 due to lack of sufficient biomass to sustain cows for a period of time. On a commercial farm, cattle may have been grazed when forage biomass yield was low or when corn was not harvested since the animals would be readily available on the farm, and stored forage resources may have been limited. However, due to the need to trailer cattle from a commercial farm for the current study, the decision was made not to transport cows if cover crop biomass was insufficient for a sustained grazing period.

**FIGURE 2:**
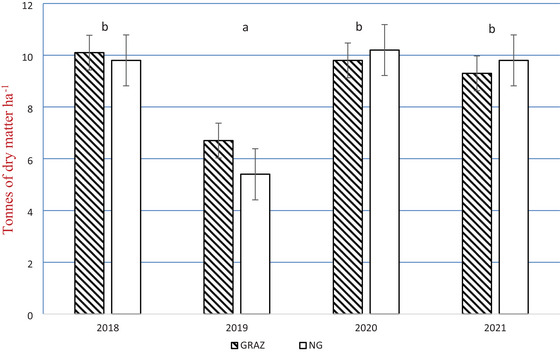
Grain yields for corn (tonnes of dry matter ha^−1^) interseeded with cereal rye that was either grazed (GRAZ) or non‐grazed (NG) by beef cattle after corn grain harvest in fall and again in early spring. Error bars are standard error of the mean. No statistical differences were found between treatments within year. There was a significant (*p* < 0.05) year effect as denoted by the superscripts letters.

Corn grain yields were typical for the northeastern United States (Curran et al., 2018; Grover et al., 2009; Youngerman et al., 2018) and did not differ between GRAZ and NG treatments or among years with the exception of 2019 when grain yields were 37%–40% lower (*p* < 0.05) than other years (with no difference between GRAZ and NG treatments; Figure 2). These results suggest that weather patterns were more influential (see Soder et al., 2024; Figures S1 and S2) on corn grain yields than interseeding cereal rye into repeated corn crops under conditions similar to those in the current study.

Not unexpectedly, there were differences among years for corn grain and pre‐grazing cereal rye yields, primarily due to variations in precipitation and temperature patterns each year. Delayed spring growth in 2019 due to dry weather resulted in minimal cereal rye growth; therefore, grazing was not conducted. Corn grain and cereal rye yields were lower in Fall 2019 due to decreased precipitation, with even greater precipitation deficits during critical growing stages for forage and corn growth and ear development. In Spring 2020, cereal rye had very little regrowth, which was a carryover effect from 2019, resulting in no grazing or biomass data collection.

In the fall, cereal rye plus corn stover provided enough forage biomass for 115–130 animal unit days ha^−1^. The only spring grazing period (2021) provided 105 animal unit days ha^−1^ of grazing. However, this grazing was cut short due to the need to prepare the sites for the next corn crop. Additional cows would have helped utilize the forage faster but were not available due to spring calving in the commercial herd.

### 3.2 Nutritive value

Pre‐ and post‐grazing nutritive values for available forage biomass (corn stover plus cereal rye) are presented in Table 3. While pre‐grazing CP, NDF, and TDN varied across years, there were no within‐year differences between GRAZ and NG treatments for any of these nutritive values. Post‐grazing nutritive quality decreased (*p* < 0.05) for the GRAZ treatments, with CP and TDN decreasing and NDF increasing in the GRAZ treatments. This is not surprising as the cattle would selectively graze the higher‐quality forage, leaving the lower‐quality (higher NDF, lower CP and TDN) forage behind (Soder et al., 2009). In the NG treatment, nutritive value decreased from the initiation to the end of grazing (3–4 weeks), which was due to degradation of the plants with the onset of early winter (McGeough et al., 2017).

**TABLE 3:** Nutritive value of aboveground dry matter (DM) forage biomass (±standard error) for grazed (GRAZ) and non‐grazed (NG) paddocks (averaged across both sites) during fall.

	Nutrient (% of DM)		
Item	CP	NDF	TDN
2018			
Fall			
Pre‐graze			
GRAZ	11.9 ± 1.08	66.2 ± 0.88	55.2 ± 2.19
NG	12.0 ± 1.09	66.5 ± 0.85	54.4 ± 2.01
Post‐graze			
GRAZ	4.6 ± 0.82a	84.1 ± 1.79a	41.3 ± 1.79a
NG	7.5 ± 0.91b	75.6 ± 1.23b	49.7 ± 1.22b
2019			
Fall			
Pre‐graze			
GRAZ	9.4 ± 0.39	72.3 ± 1.20	52.3 ± 2.89
NG	9.0 ± 0.40	73.4 ± 1.38	53.4 ± 3.01
Post‐graze			
GRAZ	4.2 ± 0.77a	85.2 ± 1.79a	40.6 ± 1.89a
NG	7.3 ± 0.81b	75.6 ± 1.23b	47.7 ± 2.40b
2020			
Fall			
Pre‐graze			
GRAZ	9.0 ± 0.36	75.1 ± 1.23	49.3 ± 3.69
NG	9.0 ± 0.54	73.8 ± 1.58	48.7 ± 4.91
Post‐graze			
GRAZ	4.4 ± 0.85a	83.7 ± 1.88a	40.6 ± 1.89a
NG	7.8 ± 0.99b	76.2 ± 1.45b	47.7 ± 2.40b
2021			
Fall			
Pre‐graze			
GRAZ	9.6 ± 0.38	75.1 ± 1.13	52.6 ± 2.01
NG	9.8 ± 0.44	73.8 ± 1.15	53.3 ± 1.89
Post‐graze	No grazing		

*Note*: Lowercase letters in columns (within nutrient) between GRAZ and NG treatments within year and sampling time (pre‐ or post‐graze) with the same letter are not significantly different (*p *< 0.05). Means averaged across both sites within season.

Abbreviations: CP, crude protein; NDF, neutral detergent fiber; TDN, total digestible nutrients.

The cereal rye was actively growing in spring in contrast to the mature, dormant cereal rye that was grazed in the fall. This resulted in nutritive quality of the cereal rye being higher in CP and TDN and lower in NDF in the spring (Table 4; Maloney et al., 2013). However, as the spring grazing season progressed, nutritive quality decreased primarily due to the cereal rye growing rapidly and becoming more mature (Liebert et al., 2023).

**TABLE 4:** Nutritive value of aboveground dry matter (DM) forage biomass (±standard error) for grazed (GRAZ) and non‐grazed (NG) paddocks (averaged across both sites) during spring.

	Nutrient (% of DM)		
Item	CP	NDF	TDN
2019			
Spring			
Pre‐graze			
GRAZ	12.2 ± 1.08	37.2 ± 0.84	71.2 ± 0.91
NG	12.2 ± 1.07	37.8 ± 0.85	72.1 ± 1.01
Post‐graze	No grazing		
2020			
Spring			
Pre‐graze			
GRAZ	12.0 ± 1.10	36.1 ± 0.80	73.3 ± 2.04
NG	12.3 ± 1.09	37.3 ± 0.85	74.4 ± 2.02
Post‐graze	No grazing		
2021			
Spring			
Pre‐graze			
GRAZ	15.7 ± 1.20	34.9 ± 0.66	74.3 ± 0.95
NG	15.5 ± 1.19	39.3 ± 0.87	72.6 ± 1.07
Post‐graze			
GRAZ	No material to harvest		
NG	7.5 ± 0.88	64.9 ± 1.56	59.6 ± 7.27

*Note*: Lowercase letters in columns (within nutrient) between GRAZ and NG treatments within year and sampling time (pre‐ or post‐graze) with the same letter are not significantly different (*p* < 0.05). Means averaged across both sites within season.

Abbreviations: CP, crude protein; NDF, neutral detergent fiber; TDN, total digestible nutrients.

### 3.3 Soil health

Grazing the cover crops over a 4‐year period had only limited impact on the measured soil health indicators. Grazing resulted in a small, but statistically significant, decrease in soil organic C and total N, while other indicators were not significantly affected by grazing (Table 5). The limited impact may reflect the short duration, limited frequency, and the low stocking density of the cattle. The lower concentrations of soil C and N with grazing likely reflect the removal of rye biomass, and possibly some corn residue, by the cattle. Subsequently, overall inputs of C and N into the GRAZ soil are somewhat lower. Moreover, grazing and subsequent deposition of manure and urine redistributes and concentrates C and N inputs into scattered small patches across the paddocks. However, soil subsamples from each paddock were composited prior to analysis, and we are unable to describe changing patterns of soil C and nutrient distribution within the paddocks. Overall, the soil health analysis suggests that short‐duration grazing of cover crops poses only limited risk to the soils. While grazing and urine deposition likely redistributed some N into small patches with elevated N content, the lower average total soil N concentration with grazing does indicate that the N fertilization rates should be more closely evaluated for the system. Additionally, soil compaction due to grazing was not addressed in the laboratory evaluation of soil health, and in‐field estimation of possible compaction by the cattle is needed.

**TABLE 5:** Comparison of soil health indicators in the upper 15 cm where rye cover crops are grazed or remain ungrazed after 6 years of cover crop planting following corn harvest for grain. Samples were obtained in the spring of 2023.

Indicator	Ungrazed	Grazed
Available water content (g g^−1^)	0.26 ± 0.01a	0.24 ± 0.01a
Aggregate stability (%)	57.40 ± 2.67a	56.83 ± 1.14a
Organic matter (%)	3.730 ± 0.10a	3.60 ± 0.99a
Organic carbon (g kg^−1^)	23.40 ± 0.73b	21.53 ± 0.41a
Total nitrogen (g kg^−1^)	2.03 ± 0.15b	1.82 ± 0.03a
Extractable protein (unitless)	6.03 ± 0.28a	5.52 ± 0.39a
Respiration (mg kg^−1^)	830 ± 70a	820 ± 30a
Active C (mg kg^−1^)	543.33 ± 36.22a	532.5 ± 29.97a
pH	6.88 ± 0.11a	6.87 ± 0.14a
Phosphorous (mg kg^−1^)	11.65 ± 3.26a	12.48 ± 4.80a
Potassium (mg kg^−1^)	155.63 ± 20.07a	167.80 ± 23.34a
Calcium (mg kg^−1^)	1892.20 ± 103.11a	1716.15 ± 59.18a
Magnesium (mg kg^−1^)	78.53 ± 5.88a	84.43 ± 16.37a
Sulfur (mg kg^−1^)	6.07 ± 0.52a	5.85 ± 0.22a
Aluminum (mg kg^−1^)	21.92 ± 3.20a	22.80 ± 3.21a
Boron (mg kg^−1^)	0.12 ± 0.01a	0.13 ± 0.02a
Copper (mg kg^−1^)	0.73 ± 0.27a	0.74 ± 0.31a
Iron (mg kg^−1^)	0.40 ± 0.08a	0.58 ± 0.14a
Manganese (mg kg^−1^)	13.72 ± 1.70a	12.96 ± 1.34a
Zinc (mg kg^−1^)	0.72 ± 0.11a	0.62 ± 0.12a

*Note*: Lowercase letters are the means and standard errors for three replicate paddocks per treatment and two sites. Values for a given measure accompanied by the same letter are not different at *p* ≤ 0.05.

### 3.4 Spatial biomass yield

**FIGURE 3:**
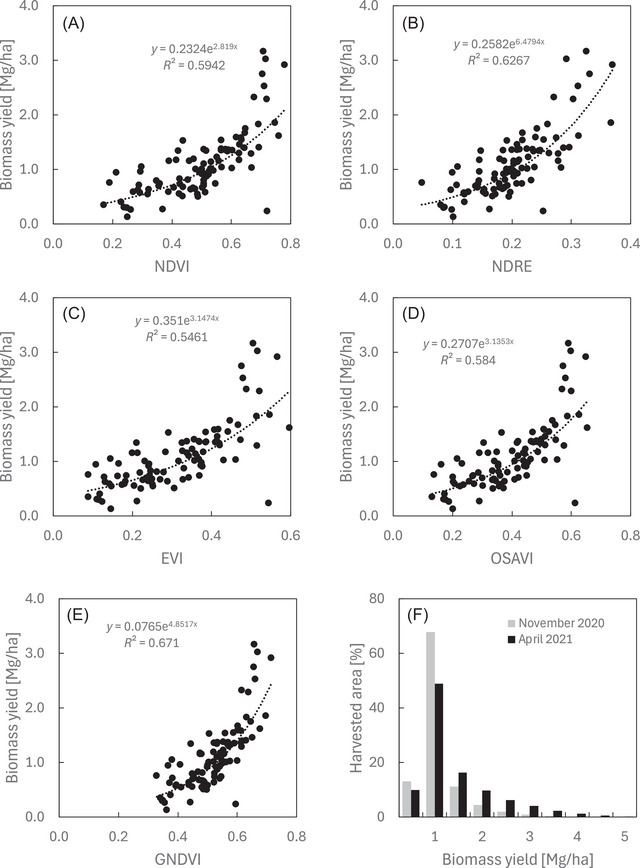
Characterization of cover crop biomass (Mg ha^−1^, rye plus weeds) using different indexes: (A) NDVI (normalized difference vegetation index), (B) NDRE (normalized difference red edge), (C) EVI (enhanced vegetation index), (D) OSAVI (optimized soil‐adjusted vegetation index), and (E) GNDVI (green normalized difference vegetation index). Exponential best‐fit line and *R* ^2^ values are presented. (F) Graph distribution of rye plus weed biomass across all paddocks before grazing in fall November 2020 and spring April 2021. Biomass was characterized using NDRE at 1 m^2^ resolution and binned into 0.5 Mg ha^−1^ increments from 0 to 5 Mg ha^−1^.

There was a range of variations in the performance of the VIs to characterize biomass yield (Figure 3). The GNDVI VI had the highest *R* ^2^ followed by NDRE > NDVI > OSAVI > EVI. Although NDRE values continued to increase with biomass yield, at ∼2 Mg ha^−1^ biomass yield, GNDVI, NDVI, EVI, and OSAVI models saturated, and the index values stopped increasing with biomass yield. Therefore, we selected the NDRE model to characterize the spatial biomass yields in the paddocks. The most common yield of biomass at 1‐m^2^ resolution was between 0.5 and 1 Mg ha^−1^ during both fall and spring periods (Figure 3F). However, in the spring, biomass yields were higher, shifting the area distribution of paddocks to higher‐yielding bins.

Many different VIs have been evaluated to characterize biomass yields (Gao et al., 2023; Prabhakara et al., 2015; Théau et al., 2021) with variable performance. There is often a threshold of saturation where the VI no longer increases with biomass yield. Prabhakara et al. (2015) found that NDVI saturated when biomass exceeds 1.5 Mg ha^−1^ for a range of cover crop species and up to 1.5 Mg ha^−1^ using NDRE (Jennewein et al., 2022), similar to our observed threshold, which approached ∼2 Mg ha^−1^ biomass yield. These findings are consistent with other work using VIs to characterize forage yield and show promise on using UAS to monitor pasture biomass yield, allowing farmers to optimize grazing patterns and improve pasture management.

### 3.5 Economics

Feed costs are the single greatest expense in livestock production system (A. Miller et al., 2001). Extending the grazing season beyond what is produced by traditional perennial cool‐season grass and legume pastures can be an economical way to feed livestock (Poore et al., 2000), as this reduces the need for more expensive hay and associated costs (Hitz & Russell, 1998). Daily feed costs for beef cows can vary widely. If we use an average stored forage cost of $2.00 day^−1^ cow^−1^ (Burdine, 2023), grazing 24 beef cows for an additional 23–26 days in the fall would result in savings in stored feed costs of $1,104–$1,248, with additional savings in feed costs in the spring if grazing is available (e.g., $1008 in Spring 2021). The cover crop seed was $174 ha^−1^ (averaged across all years of the study), and the cost of planting (including drilling, fertilizer, and herbicide) was $137 ha^−1^. This additional net income (savings in feed costs) is over and above the income from the cash crop (corn grain). Moreover, the cover crop provides many less tangible ecosystem services such as C sequestration, reduced erosion, and weed suppression (Schipanski et al., 2014).

## 4 CONCLUSIONS

Results of this study showed that late‐fall grazing of cereal rye interseeded into growing corn did not negatively impact corn grain yields with repeated corn planting. On average, the cereal rye (plus the corn stover) provided enough forage biomass for 5–130 animal unit days ha^−1^ without impairing corn grain yield. While early spring growth of the cereal rye has the potential to provide even greater forage biomass yields than fall, it is much less dependable than fall due to the potential of delayed spring growth or lack of winter precipitation under the conditions of the current study. Grazing cover crops can be an efficient way to extend the grazing season in late fall and early spring without impairing cash crop yields while providing other ecosystem services. This work supports the potential use of UAS to characterize the spatial variability of forage in paddocks to manage grazing operations.

## AUTHOR CONTRIBUTIONS

**Kathy J. Soder**: Conceptualization; data curation; formal analysis; funding acquisition; investigation; methodology; project administration; supervision; validation; visualization; writing—original draft; writing—review and editing. **Paul R. Adler**: Data curation; formal analysis; methodology; writing—review and editing. **Curtis J. Dell**: Data curation; formal analysis; writing—review and editing. **Benjamin C. Williamson**: Data curation; supervision; writing—review and editing.

## CONFLICT OF INTEREST STATEMENT

The authors declare no conflicts of interest.

## Supporting information



Supplementary Material
