# Supplementary material for: Integrating livestock and cropping systems: Interseeding cereal rye into corn for grazing
Source: J Environ Qual. 2025 Apr 25;54(5):1258–70. doi: 10.1002/jeq2.70026 (PMC12431941; doi:10.1002/jeq2.70026)
Supplement: Supplementary file 1 — Supplementary Material [file JEQ2-54-1258-s001.docx]

Supplemental Material

Supplemental Figure 1. Total precipitation and volumetric soil moisture for period when cattle grazed. Data obtained at the weather station located midway between two experimental fields.

Supplemental Figure 2: Total daily precipitation and average soil moisture at two depths for two-month periods where sampling occurred. Data was obtained from a weather station midway between the two experimental fields.
